# Associations between Arsenic in Drinking Water and Pterygium in Southwestern Taiwan

**DOI:** 10.1289/ehp.11111

**Published:** 2008-04-09

**Authors:** Wei Lin, Shu-Li Wang, Horng-Jiun Wu, Kuang-Hsi Chang, Peter Yeh, Chien-Jen Chen, How-Ran Guo

**Affiliations:** 1 Department of Environmental and Occupational Health, National Cheng Kung University, Tainan, Taiwan; 2 Division of Environmental Health and Occupational Medicine, National Health Research Institutes, Zhunan, Taiwan; 3 Institute of Environmental Medicine, College of Public Health, China Medical University Hospital, Taichung, Taiwan; 4 Department of Ophthalmology, Kaohsiung Medical University Hospital, Kaohsiung, Taiwan; 5 Department of Ophthalmology, Pingtung Hospital, Department of Health, Pingtung, Taiwan; 6 Genomics Research Center, Academia Sinica, Taipei, Taiwan; 7 Department of Occupational and Environmental Medicine, National Cheng Kung University Hospital, Tainan, Taiwan

**Keywords:** arsenic, prevalence, pterygium, ultraviolet radiation, water pollutant

## Abstract

**Background:**

Pterygium is a fibrovascular growth of the bulbar conjunctiva and underlying subconjunctival tissue that may cause blindness. The mechanism of pterygium formation is not yet fully understood, but pterygium has some tumorlike features.

**Objectives:**

The objective of this study was to evaluate the association between arsenic exposure through drinking water and the occurrence of pterygium in southwestern Taiwan.

**Methods:**

We recruited participants > 40 years of age from three villages in the arseniasis-endemic area in southwestern Taiwan (exposure villages) and four neighboring nonendemic villages (comparison villages). Each participant received an eye examination and a questionnaire interview. Photographs taken of both eyes were later graded by an ophthalmologist to determine pterygium status.

**Results:**

We included 223 participants from the exposure villages and 160 from the comparison villages. The prevalence of pterygium was higher in the exposure villages across all age groups in both sexes and increased with cumulative arsenic exposure. We found a significant association between cumulative arsenic exposure and the prevalence of pterygium. After adjusting for age, sex, working under sunlight, and working in sandy environments, we found that cumulative arsenic exposure of 0.1–15.0 mg/L-year and ≥ 15.1 mg/L-year were associated with increased risks of developing pterygium. The adjusted odds ratios were 2.04 [95% confidence interval (CI), 1.04–3.99] and 2.88 (95% CI, 1.42–5.83), respectively.

**Conclusions:**

Chronic exposure to arsenic in drinking water was related to the occurrence of pterygium, and the association was still observed after adjusting for exposures to sunlight and sandy environments.

Pterygium is a disfiguring disease that can potentially lead to blindness; complex surgery may be required to restore vision in advanced stages (Solomon et al. 2001). In general, pterygium is more prevalent in tropical and subtropical areas, where the residents have high levels of sunlight exposure (Nakaishi et al. 1997). In previous studies on general populations, the prevalence of pterygium was 17% in residents on a tropical island in Indonesia (Tan et al. 2006) and as high as 31.06% in Lima, Peru (Rojas and Malaga 1986). Because sunlight is a major risk factor, differences in sunlight exposure may lead to very different risks in people living in the same area. For example, two groups of residents of the Brazilian Amazonian rain forest with different lifestyles had prevalence rates of 36.6% and 5.0%, respectively (Paula et al. 2006). Nonetheless, even in developed countries, high prevalence rates of pterygium can still be observed. A study in Singapore found the prevalence was 6.9% among Chinese persons ≥ 40 years of age (Wong et al. 2001), and a study in Australia found a prevalence of 7.3% among men > 49 years of age (McCarty et al. 2000).

Pterygium is a winglike fibrovascular growth of the bulbar conjunctiva and underlying subconjunctival tissue of the interpalpebral fissure that may encroach onto the cornea (Jaros and DeLuise 1988). The mechanism of pterygium formation is not yet fully understood. Recent data have provided evidence implicating genetic components, anti-apoptotic mechanisms, cytokines, growth factors, extracellular matrix remodeling (through the actions of matrix metalloproteinase), immunologic mechanisms, and viral infections in the pathogenesis of this disease (Chen et al. 1994; Kwok et al. 1994). Ultraviolet light has long been recognized as a major risk factor for pterygium (McCarty et al. 2000; Moran and Hollows 1984; Wong et al. 2001); in addition to sunlight, ultraviolet radiation from welding may also cause the disease (Karai and Horiguchi 1984). Some other environmental risk factors have also been identified, including dust, low humidity, microtrauma secondary to smoke or sand, and human papilloma virus infection (Taylor et al. 1989; Varinli et al. 1994). Furthermore, pterygium has some tumorlike features, such as abnormal p53 expression and uncontrolled cell proliferation (Shimmura et al. 2000; Weinstein et al. 2002).

Arsenic, a ubiquitous element in the crust of the earth, may promote cell proliferation and is known to cause both malignant and benign tumors in human beings (Lau et al. 2004; Luster and Simeonova 2004; U.S. Environmental Protection Agency 2001). It is transported in the environment mainly by water and can be found in organic and inorganic forms in soil, air, water, and food. Humans can be exposed to arsenic from the natural environment, industrial pollution, medications, and food. Long-term exposure to ingested arsenic through drinking well water has been documented for > 50 years in an area in the southwestern coastal region of Taiwan, which is generally known as the blackfoot disease (BFD)-endemic area (BFD area) because of the prevalence of BFD, a peripheral vascular disease that may cause gangrene and thus lead to black discoloration of the affected foot (Ch’i and Blackwell 1968; Tseng 1977, 1989). It is generally believed that arsenic in drinking water is the cause of this disease. Associations have also been observed between arsenic exposure and various diseases, including cancers and other vascular diseases (Chen et al. 1985; Chiou et al. 1997, 2005; Guo 2004; Guo et al. 1997, 2001, 2004; Ibrahim et al. 2006; Wang et al. 2003; Wu et al. 1989). To evaluate the association between arsenic exposure and the development of pterygium, we conducted a study in the southwestern coastal region of Taiwan.

## Materials and Methods

We recruited participants from residents older than 40 years of age from the BFD area and a neighboring township without high arsenic levels in drinking water. Because water from shallow wells in the BFD area has high salinity, residents started consuming water from artesian wells nearly a century ago. Before the implementation of the tap water supply system in the 1960s, water from artesian wells was used for drinking and cooking. We selected the three villages with the highest prevalence of BFD as exposure villages, including Homei (prevalence of BFD: 13.6 cases/1,000), Fuhsin (9.6/1,000), and Hsinming (10.3/1,000), all in Putai Township, Chiayi County. High arsenic levels were found in the water from artesian wells in the BFD area, with the median arsenic concentration ranging from 0.70 to 0.93 mg/L in Putai Township (Chen et al. 1962). In addition, we included four villages with latitudes similar to that of Putai Township (N23.0°–N23.5°) as comparison villages, including Xinghua, Dongning, Liu’an, and Jiannan, all in Jiali Township, Tainan County (Wu et al. 1961). The median arsenic concentrations of well water in these villages were < 0.005 mg/L (Lo et al. 1977). Residents of these two groups of villages have similar racial origins, socioeconomic status, living environment, lifestyles, dietary patterns, medical facilities, and educational levels.

The recruitment procedure from residents in the exposure villages was described in detail in a previous report (Lai et al. 1994). The cumulative arsenic exposure of each participant was calculated as the sum of products, derived by multiplying the arsenic concentration in well water by the duration of water consumption during consecutive periods of living in different villages. The cumulative arsenic exposure was classified as unknown if the arsenic concentration of well water in any village where the participant had lived was unavailable. Only those who had lived in the same group of villages for > 5 years were recruited. We invited 430 residents in the exposure villages and 303 residents in the comparison villages to attend the health survey; the two groups of participants were frequency matched for age and sex.

Each participant received an eye examination conducted by an ophthalmologist and was given a questionnaire interview in 2005. The eye examination included measurement of the best corrected visual acuity and inspection of the anterior segment, using a slit lamp. After the eye examination, a well-trained technician took four photographs of both eyes from four different positions: right nasal side, right temporal side, left nasal side, and left temporal side. A well-trained interviewer conducted all the personal interviews using a standard-structure questionnaire to collect data on demographic characteristics, lifestyle factors (including alcohol drinking, cigarette smoking, and betel nut chewing), past medical history, duration of working under sunlight, and history of working in sandy environments.

An ophthalmologist who was blind to the arsenic exposure status of the participants made the diagnosis and graded pterygium using the photographs taken in the field. We adopted the three-level morphologic classification based on the visibility of the underlying episcleral blood vessels (Tan et al. 1997a), which is a useful marker of severity (Gazzard et al. 2002). In grade 1 (atrophic), episcleral vessels are clearly visible; for grade 2 (intermediate), vessels are partially visible; and in grade 3 (fleshy), vessels are wholly obscured. We defined a participant as positive for pterygium if any pterygium lesion was observed or a previous history of surgery for pterygium was confirmed for either eye. Participants with unclear photographs that led to uncertainties in the diagnosis were excluded from further data analyses.

We evaluated differences in categorical variables between groups using the chi-square test and difference in continuous variables using the two-sample *t*-test. To identify risk factors for pterygium, we applied univariate logistic regressions followed by multivariate logistic regressions. All data analyses were performed using SAS, version 8.0 (SAS Institute Inc., Cary, NC, USA) or SPSS, version 12.0 (SPSS Inc., Chicago, IL, USA), and all statistical tests were performed at the two-tailed significance level of 0.05. This study was approved by the Human Subject Review Board of the National Health Research Institutes of Taiwan, and all participants gave written informed consent prior to the study.

## Results

Our study included 223 participants from the exposure villages and 160 from the comparison villages. Among candidates from exposure villages, the mean age (63.3 vs. 64.6 years; *p* = 0.17) and the proportion of males (43.2% vs. 41.8%; *p* = 0.76) were similar between those who were included in the further analyses and who were not. In candidates from the comparison villages who were included in the analysis, the mean age was similar to that of those who were not included (62.7 vs. 62.1 years; *p* = 0.62), but this group had a higher proportion of males (46.0% vs. 30.7%; *p* = 0.01). Among the participants included in further analyses, those who lived in the exposure villages had age and sex distributions similar to those who lived in the comparison villages (Table 1).

Of the participants included in the analysis, 134 were found to be positive for pterygium during the examination, including 126 with nasal lesions and 34 with temporal lesions. Because a substantial number of them had a previous history of surgery for pterygium or more than one lesion, and thus could not be classified properly by the grading system, and because the number of pterygium patients was small in certain groups when broken down by age or other variables, we did not break down the patients by the grade in the analyses. The exposure villages had a higher overall prevalence of pterygium (Table 1), and the prevalence was higher across all age groups in both sexes (Table 2). Participants living in the exposure villages were also more likely to have worked under sunlight for ≥ 10 years (Table 1).

After adjusting for age and sex, we found that the prevalence of pterygium increased with cumulative arsenic exposures (Table 3). Although the prevalence of pterygium increased with the duration of artesian well water consumption, participants who had consumed artesian well water for ≥ 30 years had a risk similar to that of those who had consumed the water for 20–29 years.

Univariate logistic regressions showed that age, cumulative arsenic exposure, working under sunlight for ≥ 10 years, and usually or always working in sandy environments were risk factors for pterygium (Table 4). Using multivariate logistic regression, we found that all four factors were independent predictors of pterygium. After adjustment for age, sex, working under sunlight, and working in sandy environments, cumulative arsenic exposure was associated with the risk of developing pterygium.

## Discussion

In this study, we observed a positive association between the prevalence of pterygium and cumulative arsenic exposure, and we observed strong associations between the development of pterygium and two other environmental risk factors, sunlight and sand. Most epidemiology studies have found that sunlight exposure is the major risk factor for pterygium (Kwok et al. 1994; Taylor et al. 1989). One study found that the odds ratios (ORs) associated with current and previous sunlight exposures were 3.54 and 4.52, respectively (Al-Bdour and Al-Latayfeh 2004). Although the use of sunglasses offered some protection (Mackenzie et al. 1992), Al-Bdour and Al-Latayfeh (2004) did not find the effect to be statistically significant. In a study in Australia, Mackenzie et al. (1992) found the risk of pterygium to be increased several hundred-fold among participants who worked mainly on sand, compared with those who worked indoors, and attributed the risk to the high surface reflectance of ultraviolet light. We speculate that the local irritation caused by sand may also contribute to the development of pterygium.

After adjusting for sunlight and sand exposures, we found arsenic to be an independent predictor for pterygium. The prevalence we observed was higher than most previous studies of general populations living even closer to the equator (Gazzard et al. 2002; Luthra et al. 2001; Saw and Tan 1999; Wong et al. 2001), which supports our hypothesis that arsenic in drinking water is an additional risk factor. The mechanism of pterygium formation associated with arsenic exposure is unclear, but some studies found that As(III) can induce tumors in animal models (Soucy et al. 2003). Therefore, we speculate that arsenic might cause pterygium through similar mechanisms, including cell proliferation and angiogenesis. Some studies have demonstrated common features shared by pterygium and neoplasms—such as abnormal proliferation, *p53* gene overexpression, and angiogenesis—which supports the argument that pterygium is a tumorlike growth disorder (Chen et al. 1994; Dushku and Reid 1997; Dushku et al. 2001; Kria et al. 1996; Li et al. 2001; Tan et al. 1997b; Shimmura et al. 2000; Soucy et al. 2003; Weinstein et al. 2002). The predominance of nasal lesions observed in our study is compatible with findings in previous studies in Taiwan (Chen and Chuang 1976; Chen et al. 1964; Lin et al. 2006; Wang et al. 1977) and studies in other countries (Dolezalová 1977; Payrani et al. 1994).

For some study participants, we did not have data on arsenic levels in drinking water for all areas where they had lived; thus, we lacked information on cumulative arsenic exposure (the “unknown” group in Table 4). Nonetheless, the duration of drinking artesian well water was available for all participants, and we observed a positive association between the duration of drinking artesian well water and pterygium.

Long-term exposure to ingested arsenic may increase the risks of developing ischemic heart disease (Chen et al. 1996), cerebral infarction (Chiou et al. 1997; Wang et al. 2002), microcirculation abnormality (Chiou et al. 2005; Wang et al. 2003), diabetes mellitus (Tseng et al. 2000), hypertension (Chen et al. 1995), and various cancers (Cantor 2001; Chen et al. 1985, 1988, 1996; Chiou et al. 1997; Guo 2004; Guo et al. 1997, 1998, 2001, 2004; Smith et al. 1998; Tseng et al. 1996, 2000; Tsuda et al. 1995; Wu et al. 1989). Therefore, some residents of the exposure villages might not be able to participate in the study because of illness or death related to arsenic exposure, which leads to underestimations of the risks of developing pterygium. Although this will not affect our conclusion that arsenic exposure is associated with pterygium, further studies are needed to obtain more accurate risk estimates.

## Figures and Tables

**Table 1:** Comparisons between participants living in the exposure villages and comparison villages.

Variables	Exposure villages No. (%)	Comparison villages No. (%)	*p*-Value^a^
Age (years)			0.96
40–55	55 (24.7)	41 (25.6)	
56–65	70 (31.4)	51 (31.9)	
> 65	98 (43.9)	68 (42.5)	
Sex			0.41
Male	128 (57.4)	85 (53.1)	
Female	95 (42.6)	75 (46.9)	
Working under sunlight			< 0.01
< 10 years	58 (27.2)	99 (62.3)	
≥ 10 years	155 (72.8)	60 (37.7)	
Working in sandy environments			< 0.02
Never or occasionally	152 (80.4)	142 (89.2)	
Usually or always	37 (19.6)	17 (10.8)	
Pterygium			< 0.01
Positive	102 (45.7)	32 (20.0)	
Negative	121 (54.3)	128 (80.0)	

a Chi-square test.

**Table 2:** Number and prevalence (%) of pterygium cases by age, sex, and residence in exposure and comparison villages.

	Males			Females		
Age (years)	Exposure No. (%)	Comparison No. (%)	Overall No. (%)	Exposure No. (%)	Comparison No. (%)	Overall No. (%)
40–55	5 (20.8)	2 (12.5)	7 (17.5)	9 (29.0)	2 (8.0)	11 (19.6)
56–65	20 (64.5)	3 (12.0)	23 (41.1)	15 (38.5)	4 (15.4)	19 (29.2)
> 65	26 (65.0)	7 (20.6)	33 (44.6)	27 (46.6)	14 (41.2)	41 (44.6)
Total	51 (53.7)	12 (16.0)	63 (37.1)	51 (39.8)	20 (23.5)	71 (33.3)

**Table 3:** Age- and sex-adjusted odds ratios (AOR) and associated 95% CI of pterygium by indices of long-term arsenic exposure.

Arsenic exposure index	Cases	Prevalence (%)	AOR	95% CI
Cumulative arsenic exposure (mg/L-year)				
< 0.1	194	22.7	1.00	
0.1–15.0	76	44.7	3.14*	1.76–5.63
≥ 15.1	69	59.4	4.07*	2.23–7.42
Unknown	44	34.1	1.61	0.76–3.38
Duration of artesian well water consumption (years)				
0	193	22.3	1.00	
1–19	68	39.7	2.86*	1.54–5.31
20–29	76	50.0	3.31*	1.86–5.90
≥ 30	46	56.5	3.27*	1.62–6.61

* *p* < 0.05.

**Table 4:** Odds ratios (ORs), adjusted ORs (AORs), and associated 95% CIs of risk factors for developing pterygium.

Risk factor	OR	95% CI	AOR^a^	95% CI
Age (year)	1.06*	1.03–1.09	1.05*	1.02–1.09
Sex				
Female	1.00		1.00	
Male	1.18	0.77–1.80	1.24	0.74–2.08
Cumulative arsenic exposure (mg/L-year)				
< 0.1	1.00		1.00	
0.1–15.0	2.76*	1.57–4.85	2.04*	1.04–3.99
≥ 15.1	4.99*	2.78–8.97	2.88*	1.42–5.83
Unknown	1.76	0.87–3.58	1.10	0.45–2.69
Working under sunlight (years)				
< 10	1.00		1.00	
≥ 10	3.40*	2.12–5.48	2.12*	1.19–3.76
Working in sandy environments				
Never or occasionally	1.00		1.00	
Usually or always	2.99*	1.65–5.40	2.63*	1.36–5.08

a ORs adjusted for the other variables included in the multiple logistic regression model.

* *p* < 0.05.

## References

[b1-ehp0116-000952] Al-Bdour MD, Al-Latayfeh MM (2004). Risk factors for pterygium in an adult Jordanian population. Acta Ophthalmol Scand.

[b2-ehp0116-000952] Cantor KP (2001). Invited commentary: arsenic and cancer of the urinary tract. Am J Epidemiol.

[b3-ehp0116-000952] Chen C-J, Chiou H-Y, Chiang M-H, Lin L-J, Tai T-Y (1996). Dose-response relationship between ischemic heart disease mortality and long-term arsenic exposure. Arterioscler Thromb Vasc Biol.

[b4-ehp0116-000952] Chen C-J, Chung Y-C, Lin T-M, Wu H-Y (1985). Malignant neoplasms among residents of a blackfoot disease-endemic area in Taiwan: high-arsenic artesian well water and cancers. J Cancer Res.

[b5-ehp0116-000952] Chen C-J, Hsueh Y-M, Lai M-S, Shyu M-P, Chen S-Y, Wu M-M (1995). Increased prevalence of hypertension and long-term arsenic exposure. Hypertension.

[b6-ehp0116-000952] Chen C-J, Wu M-M, Lee S-S, Wang J-D, Cheng S-H, Wu H-Y (1988). Atherogenicity and carcinogenicity of high-arsenic artesian well water. Multiple risk factors and related malignant neoplasms of blackfoot disease. Arterioscler Thromb Vasc Biol.

[b7-ehp0116-000952] Chen C-W, Chuang S-S (1976). Prevalence of pterygia among plain aborigines of Paiwan tribe of Chin-Fong-Hsian, Tai-Tong-Shien. Acta Societatis Ophthalmologicae Sinicae.

[b8-ehp0116-000952] Chen C-W, Tsai Y-H, Lin C-Y, Chen S-R (1964). An epidemiologic study of pterygium among Taiwan aborigines. Acta Societatis Ophthalmologicae Sinicae.

[b9-ehp0116-000952] Chen J-K, Tsai R-J, Lin S-S (1994). Fibroblasts isolated from human pterygia exhibit transformed cell characteristics. In Vitro Cell Dev Biol Anim.

[b10-ehp0116-000952] Chen K-P, Wu H-Y, Wu T-C (1962). Epidemiologic studies on blackfoot disease in Taiwan. 3. Physicochemical characteristics of drinking water in endemic blackfoot disease areas. Memoirs College Med Natl Taiwan Univ.

[b11-ehp0116-000952] Ch’i IC, Blackwell RQ (1968). A controlled retrospective study of Blackfoot disease, an endemic peripheral gangrene disease in Taiwan. Am J Epidemiol.

[b12-ehp0116-000952] Chiou H-Y, Huang W-I, Su C-L, Chang S-F, Hsu Y-H, Chen C-J (1997). Dose-response relationship between prevalence of cerebrovascular disease and ingested inorganic arsenic. Stroke.

[b13-ehp0116-000952] Chiou J-M, Wang S-L, Chen C-J, Deng C-R, Lin W, Tai TY (2005). Arsenic ingestion and increased microvascular disease risk: observations from the south-western arseniasis-endemic area in Taiwan. Int J Epidemiol.

[b14-ehp0116-000952] Dolezalová V (1977). Is the occurrence of a temporal pterygium really so rare?. Ophthalmologica.

[b15-ehp0116-000952] Dushku N, John MK, Schultz GS, Reid TW (2001). Pterygia pathogenesis: corneal invasion by matrix metalloproteinase expressing altered limbal epithelial basal cells. Arch Ophthalmol.

[b16-ehp0116-000952] Dushku N, Reid TW (1997). P53 expression in altered limbal basal cells of pingueculae, pterygia, and limbal tumors. Curr Eye Res.

[b17-ehp0116-000952] Gazzard G, Saw SM, Farook M, Koh D, Widjaja D, Chia SE (2002). Pterygium in Indonesia: prevalence, severity and risk factors. Br J Ophthalmol.

[b18-ehp0116-000952] Guo H-R (2004). Arsenic level in drinking water and mortality of lung cancer (Taiwan). Cancer Causes Control.

[b19-ehp0116-000952] Guo H-R, Chiang H-S, Hu H, Lipsitz SR, Monson RR (1997). Arsenic in drinking water and incidence of urinary cancers. Epidemiology.

[b20-ehp0116-000952] Guo H-R, Lipsitz SR, Hu H, Monson RR (1998). Using ecological data to estimate a regression model for individual data: the association between arsenic in drinking water and incidence of skin cancer. Environ Res.

[b21-ehp0116-000952] Guo H-R, Wang N-S, Hu H, Monson RR (2004). Cell type specificity of lung cancer associated with arsenic ingestion. Cancer Epidemiol Biomarkers Prev.

[b22-ehp0116-000952] Guo H-R, Yu H-S, Hu H, Monson RR (2001). Arsenic in drinking water and skin cancers: cell-type specificity (Taiwan, ROC). Cancer Causes Control.

[b23-ehp0116-000952] Ibrahim D, Froberg B, Wolf A, Rusyniak DE (2006). Heavy metal poisoning: clinical presentations and pathophysiology. Clin Lab Med.

[b24-ehp0116-000952] Jaros PA, DeLuise VP (1988). Pingueculae and pterygia. Surv Ophthalmol.

[b25-ehp0116-000952] Karai I, Horiguchi S (1984). Pterygium in welders. Br J Ophthalmol.

[b26-ehp0116-000952] Kria L, Ohira A, Amemiya T (1996). Immunohistochemical localization of basic fibroblast growth factor, platelet derived growth factor, transforming growth factor-beta and tumor necrosis factor-alpha in the pterygium. Acta Histochem.

[b27-ehp0116-000952] Kwok SK, Ho PC, Leung SF, Sonal KF (1994). Surgical result of radiation-induced cataract in Chinese patients with nasopharyngeal carcinoma. Dev Ophthalmol.

[b28-ehp0116-000952] Lai M-S, Hsueh Y-M, Chen C-J, Shyu M-P, Chen S-Y, Kuo T-L (1994). Ingested inorganic arsenic and prevalence of diabetes mellitus. Am J Epidemiol.

[b29-ehp0116-000952] Lau AT, Li M, Xie R, He QY, Chiu JF (2004). Opposed arsenite-induced signaling pathways promote cell proliferation or apoptosis in cultured lung cells. Carcinogenesis.

[b30-ehp0116-000952] Li DQ, Lee SB, Gunja-Smith Z, Liu Y, Solomon A, Meller D (2001). Over-expression of collagenase (MMP-1) and stromelysin (MMP-3) by pterygium head fibroblasts. Arch Ophthalmol.

[b31-ehp0116-000952] Lin S-F, Tsai R-K, Tung I-C, Sheu M-M (2006). An epidemiologic study of pterygium in middle-aged and elderly aboriginal populations of the Tao Tribe of Orchid Island in Taiwan. Tzu Chi Med J.

[b32-ehp0116-000952] Lo M-C, Hsen Y-C, Lin B-K (1977). Second Report on the Investigation of Arsenic Content in Underground Water in Taiwan.

[b33-ehp0116-000952] Luster MI, Simeonova PP (2004). Arsenic and urinary bladder cell proliferation. Toxicol Appl Pharmacol.

[b34-ehp0116-000952] Luthra R, Nemesure BB, Wu SY, Xie SH, Leske MC (2001). Frequency and risk factors for pterygium in the Barbados Eye Study. Arch Ophthalmol.

[b35-ehp0116-000952] Mackenzie FD, Hirst LW, Battistutta D, Green A (1992). Risk analysis in the development of pterygia. Ophthalmology.

[b36-ehp0116-000952] McCarty CA, Fu CL, Taylor HR (2000). Epidemiology of pterygium in Victoria, Australia. Br J Ophthalmol.

[b37-ehp0116-000952] Moran DJ, Hollows FC (1984). Pterygium and ultraviolet radiation: a positive correlation. Br J Ophthalmol.

[b38-ehp0116-000952] Nakaishi H, Yamamoto M, Ishida M, Someya I, Yamada Y (1997). Pingueculae and pterygia in motorcycle policemen. Ind Health.

[b39-ehp0116-000952] Paula JS, Thorn F, Cruz AA (2006). Prevalence of pterygium and cataract in indigenous populations of the Brazilian Amazon rain forest. Eye.

[b40-ehp0116-000952] Payrani SB, Scott WP, Wells JW, Johnson DW, Chobe RJ, Kuruvilla A (1994). Management of pterygium with surgery and radiation therapy. The North Florida Pterygium Study Group. Int J Radiat Oncol Biol Phys.

[b41-ehp0116-000952] Rojas JR, Malaga H (1986). Pterygium in Lima, Peru. Ann Ophthalmol.

[b42-ehp0116-000952] Saw SM, Tan D (1999). Pterygium: prevalence, demography and risk factors. Ophthalmic Epidemiol.

[b43-ehp0116-000952] Shimmura S, Ishioka M, Hanada K, Shimazaki J, Tsubota K (2000). Telomerase activity and p53 expression in pterygia. Invest Ophthalmol Vis Sci.

[b44-ehp0116-000952] Smith AH, Goycolea M, Haque R, Biggs ML (1998). Marked increase in bladder and lung cancer mortality in a region of northern Chile due to arsenic in drinking water. Am J Epidemiol.

[b45-ehp0116-000952] Solomon A, Pires RT, Tseng SC (2001). Amniotic membrane transplantation after extensive removal of primary and recurrent pterygia. Ophthalmology.

[b46-ehp0116-000952] Soucy NV, Ihnat MA, Kamat CD, Hess L, Post MJ, Klei LR (2003). Arsenic stimulates angiogenesis and tumorigenesis *in vivo*. Toxicol Sci.

[b47-ehp0116-000952] Tan CS, Lim TH, Koh WP, Liew GC, Hoh ST, Tan CC (2006). Epidemiology of pterygium on a tropical island in the Riau Archipelago. Eye.

[b48-ehp0116-000952] Tan DT, Chee SP, Dear KB, Lim AS (1997a). Effect of pterygium morphology on pterygium recurrence in a controlled trial comparing conjunctival autografting with bare sclera excision. Arch Ophthalmol.

[b49-ehp0116-000952] Tan DT, Lim AS, Goh HS, Smith DR (1997b). Abnormal expression of the p53 tumor suppressor gene in the conjunctiva of patients with pterygium. Am J Ophthalmol.

[b50-ehp0116-000952] Taylor HR, West SK, Rosenthal FS, Munoz B, Newland HS, Emmett EA (1989). Corneal changes associated with chronic UV irradiation. Arch Ophthalmol.

[b51-ehp0116-000952] Tseng C-H, Chong C-K, Chen C-J, Tai T-Y (1996). Dose-response relationship between peripheral vascular disease and ingested inorganic arsenic among residents in blackfoot disease endemic villages in Taiwan. Atherosclerosis.

[b52-ehp0116-000952] Tseng C-H, Tai T-Y, Chong C-K, Tseng C-P, Lai M-S, Lin B-J (2000). Long-term arsenic exposure and incidence of non-insulin-dependent diabetes mellitus: a cohort study in arseniasis-hyperendemic villages in Taiwan. Environ Health Perspect.

[b53-ehp0116-000952] Tseng W-P (1977). Effects and dose-response relationships of skin cancer and blackfoot disease with arsenic. Environ Health Perspect.

[b54-ehp0116-000952] Tseng W-P (1989). Blackfoot disease in Taiwan: a 30-year follow-up study. Angiology.

[b55-ehp0116-000952] Tsuda T, Babazono A, Yamamoto E, Kurumatani N, Mino Y, Ogawa T (1995). Ingested arsenic and internal cancer: a historical cohort study followed for 33 years. Am J Epidemiol.

[b56-ehp0116-000952] U.S. Environmental Protection Agency (2001). National primary drinking water regulations. Arsenic and clarifications to compliance and new source contaminants monitoring. Final rule. Fed Reg.

[b57-ehp0116-000952] Varinli S, Varinli I, Köksal Erkisi M, Doran F (1994). Human papillomavirus in pterygium. Cent Afr J Med.

[b58-ehp0116-000952] Wang C-H, Jeng J-S, Yip P-K, Chen C-L, Hsu L-I, Hsueh Y-M (2002). Biological gradient between long-term arsenic exposure and carotid atherosclerosis. Circulation.

[b59-ehp0116-000952] Wang H-Z, Chuang S-S, Chen C-W (1977). The prevalence of pterygia in Pescadores Island. Acta Societatis Ophthalmologicae Sinicae.

[b60-ehp0116-000952] Wang S-L, Chiou J-M, Chen C-J, Tseng C-H, Chou W-L, Wang C-C (2003). Prevalence of non-insulin-dependent diabetes mellitus and related vascular diseases in southwestern arseniasis-endemic and nonendemic areas in Taiwan. Environ Health Perspect.

[b61-ehp0116-000952] Weinstein O, Rosenthal G, Zirkin H, Monos T, Lifshitz T, Argov S (2002). Overexpression of p53 tumor suppressor gene in pterygia. Eye.

[b62-ehp0116-000952] Wong TY, Foster PJ, Johnson GJ, Seah SK, Tan DT (2001). The prevalence and risk factors for pterygium in an adult Chinese population in Singapore: the Tanjong Pagar survey. Am J Ophthalmol.

[b63-ehp0116-000952] Wu H-Y, Chen K-P, Tseng W-P, Hsu C-L (1961). Epidemiologic studies on blackfoot disease. I. Prevalence and incidence of the disease by age, sex, year, occupation and geographical distribution. Mem College Med Natl Taiwan Univ.

[b64-ehp0116-000952] Wu M-M, Kuo T-L, Hwang Y-H, Chen C-J (1989). Dose-response relation between arsenic concentration in well water and mortality from cancers and vascular diseases. Am J Epidemiol.

